# Excretable Lanthanide Nanoparticle for Biomedical Imaging and Surgical Navigation in the Second Near‐Infrared Window

**DOI:** 10.1002/advs.201902042

**Published:** 2019-10-04

**Authors:** Daifeng Li, Shuqing He, Yifan Wu, Jianqiang Liu, Qiang Liu, Baisong Chang, Qing Zhang, Zhanhong Xiang, Ying Yuan, Chao Jian, Aixi Yu, Zhen Cheng

**Affiliations:** ^1^ Department of Orthopedics Trauma and Microsurgery Zhongnan Hospital of Wuhan University Wuhan Hubei 430071 China; ^2^ Molecular Imaging Program at Stanford (MIPS) Bio‐X Program and Department of Radiology Canary Center at Stanford for Cancer Early Detection Stanford University Stanford CA 94305‐5344 USA; ^3^ Academy for Advanced Interdisciplinary Studies and Department of Biomedical Engineering Southern University of Science and Technology (SUSTech) Shenzhen 518055 China; ^4^ Department of Orthopedics The Fourth Hospital of Jinan Jinan Shandong 250031 China

**Keywords:** excretable lanthanide nanoparticles, liposomes, second near‐infrared window, surgical navigation, vascular disorders imaging

## Abstract

Recently, various second near‐infrared window (NIR‐II, 1000–1700 nm) fluorophores have been synthesized for in vivo imaging with nonradiation, high resolution, and low autofluorescence. However, most of the NIR‐II fluorophores, especially inorganic nanoprobes, are mainly retained in the reticuloendothelial system (RES) such as the liver and spleen, leading to long‐term safety concerns. Herein, a type of lanthanide‐based excretable NIR‐II nanoparticle, RENPs@Lips, which can be quickly cleared out of body after intravenous administration with half‐lives of 23.0 h for the liver and 14.9 h for the spleen, is reported. Interestingly, over 90% of RENPs@Lips can be excreted through a hepatobiliary system within 72 h postinjection. The moderate blood half‐time (*T*_1/2_ = 17.96 min) allows for multifunctional applications in delineating the hemodynamics of vascular disorders (artery thrombosis, ischemia, and tumor angiogenesis) and monitoring blood perfusion in response to acute ischemia. In addition, RENPs@Lips exhibit high performance in identifying orthotopic tumor vessels intraoperatively and embolization surgery under NIR‐II imaging navigation. Moreover, excellent signal‐to‐background ratio (SBR) is successfully achieved to facilitate sentinel lymph nodes biopsy (SLNB) with tumor‐bearing mice. The high biocompatibility, favorable excretability, and outstanding optical properties warrant RENPs@Lips as novel promising NIR‐II nanoparticles for future applications and translation into an interdisciplinary amalgamation of research in diverse fields.

## Graphical abstract

Here, a type of liposome‐coated lanthanide second near‐infrared window (NIR‐II) nanoparticle, RENPs@Lips, is reported, demonstrating that over 90% can be excreted through the hepatobiliary system within 72 h postinjection. Further work demonstrates the capability of delineating the hemodynamics of vascular disorders and accomplishing surgery under NIR‐II imaging navigation, showing great potential of excretable RENPs@Lips for future applications and translations in diverse fields.

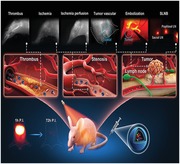

## 1 Introduction

Over the past decades, the establishment of diverse preclinical animal models significantly contributes to the delineation of physiological and pathological mechanisms, providing indispensable platforms to facilitate the development of novel therapeutic strategies.^1^, ^2^, ^3^, ^4^ Therefore, it requires effective imaging tools to assess the progression of diseases and their therapeutic response. Unfortunately, traditional imaging techniques (magnetic resonance imaging (MRI), positron emission tomography (PET), and computed tomography (CT)) are suboptimal to achieve such goals ascribed to poor sensitivity, limited resolution, exposure to radiation, and high expense.^5^, ^6^ As a promising candidate of imaging modality with respect to noninvasive and nonradiation, optical imaging has been an area of intense focus in real‐time visualization and monitoring the dynamic pathological processes of diseases.^7^, ^8^, ^9^, ^10^, ^11^ To date, compared to fluorescence imaging in the visible and near‐infrared wavelengths (<900 nm), deep tissue imaging in the second near‐infrared window (NIR‐II, 1000–1700 nm) has benefited from negligible tissue autofluorescence and scattering, leading to superior improvements in higher resolution and fidelity. Thus, it has emerged as the next‐generation imaging technique for in vivo biomedical imaging.^12^, ^13^, ^14^, ^15^, ^16^, ^17^

Recently, tremendous efforts have been devoted to design and synthesize NIR‐II fluorophores with high performance, including organic molecules^16^, ^17^, ^18^, ^19^ and inorganic nanoparticles.^20^, ^21^, ^22^, ^23^, ^24^ However, tedious synthetic procedure and low quantum yield of organic molecules are still the main chemical impediments that make them lie behind the inorganic counterparts.^13^ Compared with several other NIR‐II inorganic fluorophores such as quantum dots (QDs) and single‐walled carbon nanotubes (SWNTs), growing interests have been focused to rare earth–doped nanoparticles (RENPs) because of their large Stokes shifts, narrow and multipeak emission profiles, negligible excitation–emission band overlap, and excellent photostability.^23^, ^24^, ^25^, ^26^, ^27^, ^28^, ^29^, ^30^, ^31^ Nevertheless, like most of reported NIR‐II inorganic nanoparticles, long retention in reticuloendothelial system (RES) and inability to clear from living body lead to the existing dilemmas in the RENPs, which also raise the potential safety concerns and greatly hamper their future broad biomedical applications and translation.^15^, ^31^, ^32^, ^33^, ^34^, ^35^, ^36^

Current researches have elucidated that the major excretory routes including renal pathway (bladder and urine) and hepatobiliary process (bile to feces) of nanoparticles are determined primarily by the size and surface charge of the nanoparticles.^33^, ^37^ However, the majority of RENPs do not satisfy the ultrasmall threshold of renal clearance (6–8 nm). Thus, surface modification of RENPs is crucial to improve their excretability from the RES system (liver and spleen) and reduce potential in vivo toxicity. PEGylation step can prolong the blood circulation time and help nanoparticles to escape from accumulation in the RES system.^38^, ^39^, ^40^, ^41^ However, there are few reports depicting the excretion of NIR‐II lanthanide‐based fluorophores with sizes beyond threshold of renal clearance. Recently, our group has synthesized novel RENPs consisting of β‐phase NaYF_4_/Nd 7%@NaYF_4_ using the thermal decomposition method. These RENPs exhibit dual fluorescent emission at 1064 and 1345 nm. They were further coated with 1,2‐distearoyl‐*sn*‐glycero‐3‐phosphoethanolamine with conjugated methoxyl poly(ethylene glycol) (DSPE‐mPEG, 5 kDa) to generate the NIR‐II probe, RENPs@DSPE‐mPEG, and showed inherent affinity to bone, which were successfully employed for in vivo imaging of skeletal system and blood vessels with high performance.^42^ Although it has been demonstrated that RENPs@DSPE‐mPEG could be eventually metabolized in vivo after intravenous administration, it shows long retention in the RES system and some normal organs, for example, the excretion half‐lives of the nanoparticle for liver, spleen, and bone is 52, 175, and 250 h, respectively, which may impact the ability of detection abnormalities in these organs and also cause concerns on the potential toxicity to RES system and bone marrow.^42^

**Scheme 1:**
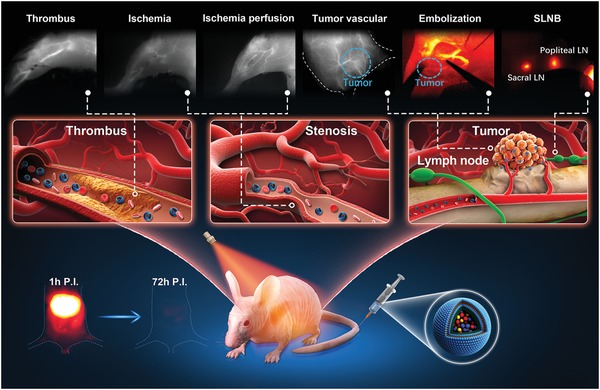
Schematic illustration of excretable lanthanide nanoparticle for multifunctional biomedical imaging and surgical navigation in the second near‐infrared window.

Liposomes have been considered to be Food and Drug Administration (FDA)‐approved carriers in drug delivery, and they are well known for their natural amphiphilic molecules' composition with favorable biocompatibility and biodegradability.^38^, ^43^ Moreover, increasing evidences demonstrate PEGylated phospholipid micelle can greatly address the toxicity issue and accelerate the clearance of fluorophores through the hepatobiliary route.^44^, ^45^, ^46^ Therefore, inspired by promising results of RENPs@DSPE‐mPEG, more researches on surface modification of the lanthanide‐based fluorophores to facilitate their excretion are considered to be important. Herein, we mixed 1,2‐dipalmitoyl phosphatidylcholine (DPPC), cholesterol (Chol), and PEGylated lipid (1,2‐distearoyl‐*sn*‐glycero‐3‐phosphoethanolamine‐*N*‐[methoxy‐(polyethylene glycol)‐2000], DSPE‐PEG2000) at a molar ratio of 77.5:20:2.5 to synthesize the liposome and then to use the liposome to further coat the NIR‐II lanthanide fluorophore RENPs. The resulted RENPs@Lips displayed significantly enhanced intravenous excretability and colloidal stability as well as reduced residence time in the RES system. Notably, over 90% of RENPs@Lips were removed from liver within only 72 h after intravenous administration. Compared to RENPs@DSPE‐mPEG, it showed much faster in vivo clearance and shorter half‐lives (17.96 vs 20.56 min for blood, 23.0 vs 52 h for liver, and 14.9 vs 175 h for spleen, respectively). Interestingly, RENPs@Lips did not show obvious accumulation to bone which may contribute to less retention in skeletal system and quicker intravenous clearance in vivo. The high performance in biocompatibility both in vitro and in vivo greatly reduced potential safety concerns. In addition, RENPs@Lips demonstrated the capability of delineating the hemodynamics of vascular disorders (artery thrombosis, ischemia, and tumor angiogenesis) and monitoring the blood perfusion in response to acute ischemia. Furthermore, the excellent signal‐to‐background ratio (SBR) implicated capable of accomplishing orthotopic osteosarcoma embolization surgery intraoperatively and sentinel lymph nodes biopsy (SLNB) with tumor‐bearing mice under NIR‐II imaging navigation (**Scheme** 1). Overall, these findings proved that the RENPs@Lips with high biocompatibility, favorable intravenous excretability, and outstanding photochemical properties are suitable to assess and monitor the physiological and pathological processes preclinically and boost their future clinical translation.

## 2 Results and Discussion

### 2.1 Synthesis and Characterization

**Figure 1:**
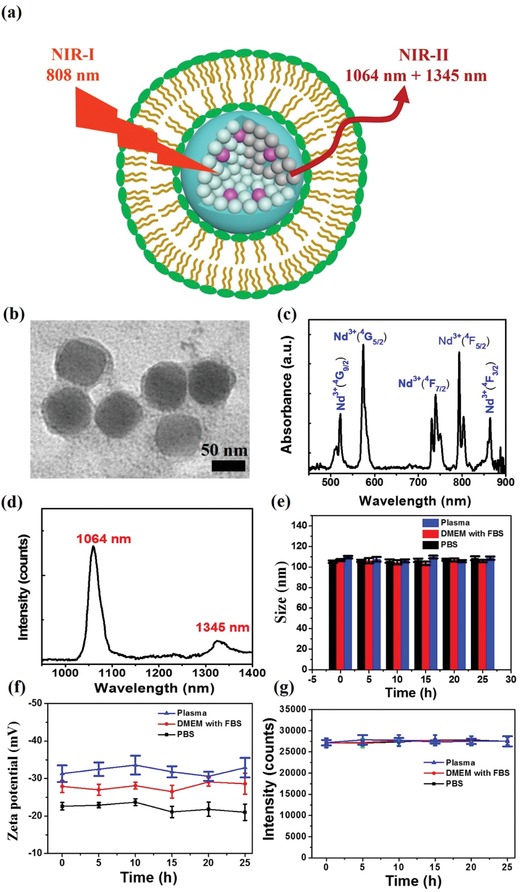
Design and synthesis of RENPs@ Lips. a) Schematic structure illustration of RENPs@Lips. b) TEM image of RENPs@Lips. c,d) Absorbance and fluorescence emission of RENPs@Lips, demonstrating multiple absorbance peaks including one at 800 nm and dual emission peaks at 1064 and 1345 nm. The fluorescence emission spectrum was obtained using an 808 nm excitation laser. e) Hydrodynamic size of nanoparticle was measured by dynamic light scattering using Zetasizer Nano ZS90. f) Zeta potential of the nanoparticles in DMEM/FBS, plasma, and PBS (as the control) for 25 h at 37 °C. g) Photostability of the NIR‐II probe in DMEM/FBS, plasma, and PBS (as the control) for 25 h at 37 °C under continuous 808 nm exposure at a power density of 0.33 W cm^−2^.

**Figure** 1a showed the schematic of the RENPs@Lips. First, NIR‐II nanoparticles RENPs were synthesized using the thermal decomposition method in high boiling point organic solvent oleic acid. The original oleic acid–coating RENPs were hydrophobic, and surface modification was needed for improving the dispersion of RENPs in water. So hydrophobic RENPs were coated with liposomes. The transmission electron microscopy (TEM) result of RENPs@Lips demonstrated monodisperse (Figure S1, Supporting Information) and homogeneous size 72.2 ± 1.6 nm (coating thickness = 4.3 ± 0.8 nm; Figure 1b). In addition, the P and O elements mapping demonstrated that liposome was coated on the RENPs successfully (Figure S2, Supporting Information). Multiabsorption peak of the RENPs@Lips was observed in the UV–vis/NIR absorption spectrum (Figure 1c). RENPs@Lips exhibited dual emission at 1064 and 1345 nm (Figure 1d) with large stokes shifts (264 and 545 nm, respectively). RENPs' emission wavelengths are highly dependent on several factors such as its elements composition and percentage of doping materials. Moreover, RENPs can produce multiple emission peaks in NIR regions. All these features make it is possible to tune the photochemical properties of RENPs for multiplexing imaging purpose. The quantum yield of RENPs@Lips was calculated to be 7.9% at 1064 nm and 4.1% at 1345 nm under 808 nm excitation (IR26 as a reference 0.5% in 1,2‐dichloroethane (DCE); Figure S3, Supporting Information).^17^ The physicochemical stability of the nanoparticles was assessed with two methods including in biological medium, Dulbecco's Modified Eagle Medium (DMEM), supplemented with 10% fetal bovine serum (FBS) and in plasma in vivo. As shown in Figure 1e, the mean hydrodynamic size of the nanoparticle is 105 ± 5.2 nm and remains the same over long time (25 h) without aggregation. In addition, the zeta potential of the nanoparticle both in DMEM+FBS and in plasma in vivo was higher than that in phosphate‐buffered saline (PBS, pH 7.4, 10 × 10^−3^ m; Figure 1f), confirming the good physicochemical stability and will not be aggregated in blood in vivo. In addition, the optical stability of the nanoprobes in DMEM/FBS and in plasma was evaluated with 808 nm laser (0.33 W cm^−2^). The NIR‐II fluorescent emission intensity of the nanoprobe did not decrease, suggesting high photostability of the NIR‐II probes (Figure 1g). The excellent physicochemical and optical performance proved that RENPs@Lips were suitable for NIR‐II imaging in vivo.

### 2.2 Toxicity Assays

**Figure 2:**
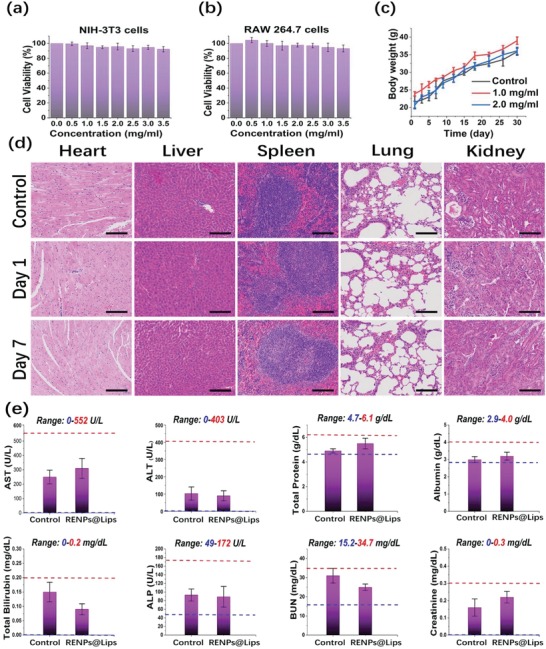
Toxicity assays of RENPs@Lips. a,b) MTT analyses of NIH 3T3 and RAW 264.7 cells demonstrated no apparent cytotoxicity after 48 h of incubation in vitro. c) All mice with different doses treatment showed no significant difference in weight changes during the entire observation period of 30 days (*n* = 3). d) Histological analysis of major organs (heart, liver, spleen, lung, and kidneys), and no obvious hydropic damage or necrotic lesions were revealed compared to the normal controlled group at 1 and 7 days after intravenous administration, respectively. Scale bar: 100 µm. e) Blood chemistry profiles including ALT, AST, TP, albumin, TB, ALP, BUN, and Cr. None of these blood biomarkers was significantly altered compared with the control groups, suggesting the absence of liver or kidney toxicity.

Increasing concerns in NIR‐II fluorophores mandate high in vivo biocompatibility of the nanoprobe prepared. As the FDA‐approved carriers in drug delivery, liposomes have been well known for their favorable biocompatibility, which can greatly address the toxicity issue.^38^, ^43^ To evaluate the potential toxicity of RENPs@Lips, we first performed a standard 3‐(4,5‐dimethylthiazol‐2‐yl)‐2,5‐diphenyl tetrazolium bromide (MTT) assay on the embryonic fibroblast cell NIH 3T3 and microphage cell RAW 264.7 and the results are shown in **Figure** 2a,b. It was observed that RENPs@Lips exhibited no apparent cytotoxicity to both normal cell lines even at the concentration up to 3.5 mg mL^−1^, suggesting their low cytotoxicity in vitro. Furthermore, we examined the in vivo toxicity in normal nude mice through intravenous injection of the nanoprobe. All the mice exhibited no signs of abnormal behavior and/or clinical symptoms of toxicity as well as no significant difference in weight changes during the entire observation period of 30 days at different injection doses (Figure 2c). In addition, the vital organs of the mice were harvested at different time points. Histological analysis of them demonstrated no obvious hydropic damage or necrotic lesions compared to the normal control groups at 1 and 7 days after intravenous administration, respectively (Figure 2d). Meanwhile, blood chemistry profiles including alanine aminotransferase (ALT), aspartate aminotransferase (AST), total protein (TP), albumin, total bilirubin (TB), alkaline phosphatase (ALP), blood urea nitrogen (BUN), and creatinine (Cr) revealed none of these blood biomarkers was significantly altered compared with the control groups, suggesting the absence of liver or kidney toxicity (Figure 2e). The high biocompatibility and well toleration of the nanoparticles indicated that RENPs@Lips were appropriate for NIR‐II deep tissue imaging in vivo.

### 2.3 In Vivo Pharmacokinetics

**Figure 3:**
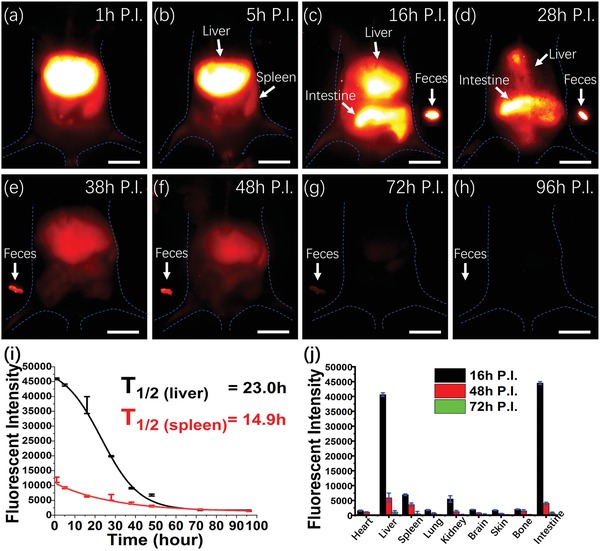
In vivo pharmacokinetics. a–h) Selected time points (totally for 96 h) from in vivo NIR‐II imaging of whole‐body level and ex vivo representative feces samples after intravenous administration of RENPs@Lips. Scale bar: 10 mm. i,j) Quantitation analysis of NIR‐II fluorescence intensity in vivo (liver and spleen region) and ex vivo of major organs (heart, liver, spleen, lung, kidney, brain, intestine, skin, and bone) at different time points. Half‐lives of liver and spleen were calculated as 23.0 and 14.9 h, respectively.

The slow metabolism rates (months) of most inorganic‐based fluorophores dramatically cause potential damage to the RES system and present the main obstacles for further clinical translations. To investigate the pharmacokinetics of RENPs@Lips in vivo, the nude mice (*n* = 3) and C57BL/6 mice (*n* = 3) were employed. NIR‐II imaging of whole body was performed shortly after RENPs@Lips (200 µL, 1.0 mg mL^−1^) were intravenously injected, and fluorescent signals were mainly observed in the liver, spleen, and intestines (**Figure** 3a–i). Notably, the fluorescent signal of intestines sharply increased within 16 h and then declined over time and reached to very low level at 72 h postinjection (Figure 3b,c). Meanwhile, we collected the feces and imaged ex vivo under NIR‐II setup as well as analyzed inductively coupled plasma mass spectrometry (ICP‐MS) level.^42^ Not surprisingly, intense signals of feces were captured, implicating the excretion of RENPs@Lips through hepatobiliary route from liver to intestines (bile to feces). It is noteworthy that the fluorescent signals of vital RES system organs (liver and spleen) exhibited a quick decline at 28 h and very weak signals could be visible in the next 3 days (Figure 3d–h). According to Figure 3i,j, the in vivo half‐lives of RENPs@Lips in liver and spleen were calculated as 23.0 and 14.9 h, respectively. Especially at 72 h after intravenous administration, the fluorescence accumulation in the liver dropped over 90%. Finally, the mice were sacrificed, and vital organs were harvested to analyze the fluorescence biodistribution ex vivo and ICP‐MS data at different time points.^42^ (Figure 3j; Figure S4 and Table S1, Supporting Information) In addition, ICP‐MS analysis of liver at 72 h exhibited an over 98% decline compared to the level of 16 h postinjection, and ICP‐MS result of spleen at 72 h reached to the normal level compared to the control. These results showed high consistent with our imaging data in vivo (Figure 3i). Moreover, kidney also showed some fluorescent signal at 16 h but extremely low signal at 48 h postinjection, which was also consistent with their ICP‐MS analysis. Fluorescent signal of urine was not observed, and ICP‐MS of urine did not exhibit increase compared to the control group. These results demonstrated that the RENPs@Lips were very stable without any decomposition and ions leakage. Currently, increasing evidence demonstrates that PEGylated phospholipid micelle can effectively escape the capture in the RES system and promote the excretion of fluorophores through the hepatobiliary route.^44^, ^45^, ^46^ Especially compared to the excretion rate of our previous RENP@DSPE‐mPEG (without liposome coating), it showed much faster in vivo clearance and shorter half‐lives due to liposome coating^42^ (17.96 vs 20.56 min for blood, 23.0 vs 52 h for liver, and 14.9 vs 175 h for spleen, respectively; Table S2, Supporting Information). Interestingly, after the strategy of coating liposome, RENPs@Lips did not show obvious accumulation to bone (Figure 3a–h; Figure S4 and Tables S1 and S2, Supporting Information), which may contribute to less retention in the skeletal system and quicker clearance in vivo. All these findings demonstrated that over 90% of the RENPs@Lips were excreted mainly through hepatobiliary route within 72 h, indicating that the most difficult challenge in RENPs' concerns could be addressed. Also, our strategy of liposome coating to RENPs suggested attainable biosafety to adopt to widespread preclinical studies and highlighted the promising translational potential of RENPs@Lips.

### 2.4 NIR‐II Imaging of the Circulatory System

**Figure 4:**
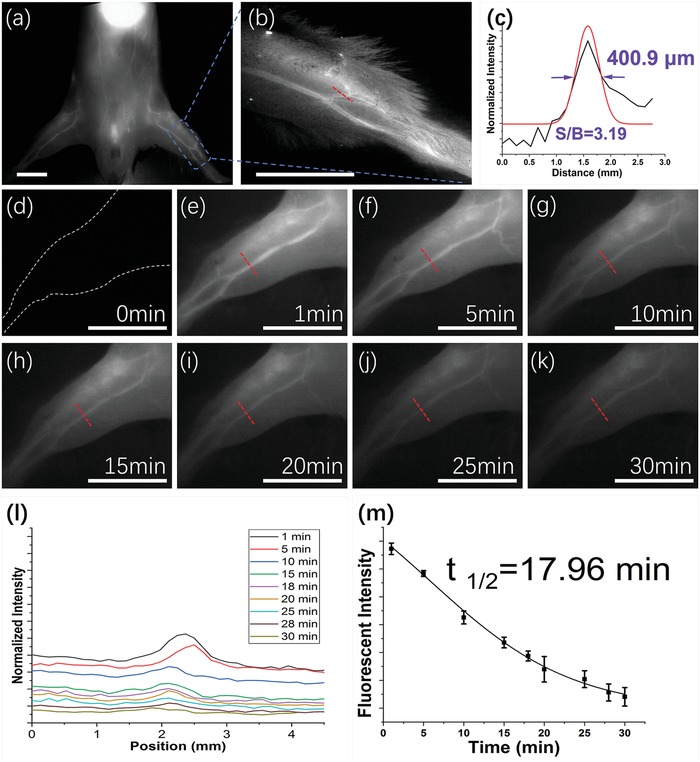
NIR‐II imaging of circulatory system. a) In vivo NIR‐II imaging of circulatory system at whole‐body level. b,c) Magnification of hind limb vascular and analysis of SBR as well as the vessel FWHM width; red lines in panel (b). d–k) NIR‐II imaging of femoral artery for 30 min after intravenous administration of RENPs@Lips. l) Normalized NIR‐II intensity of femoral artery during period of panels (d)–(k). m) Integrated NIR‐II intensity of femoral artery during period of panels (d)–(k) showed a blood half‐time of 17.96 min. Scale bar: 10 mm.

The dysfunction of blood circulatory system involves in various pathological processes and has a major medical impact. Optical imaging provides a promising alternative modality in blood circulatory system with nonradiation and high resolution. Relying on the attractive pharmacokinetics above, we first investigated the NIR‐II deep tissue imaging of blood circulation on normal C57BL/6 mice (*n* = 3). Thanks to the outstanding optics properties, NIR‐II signal of blood flow was recorded immediately after intravenous administration of RENPs@Lips and the vessel structure was clearly identified at the whole‐body level (**Figure** 4a). Moreover, a 2.5× camera lens was performed to detect the high‐magnification NIR‐II imaging of hind limb vessels (Figure 4b). The Gaussian‐fitted full width at half maximum (FWHM) was 400.9 µm (Figure 4c) measured by plotting the red line of vessels profiles in Figure 6b and the SBR was 3.19 (Figure 4c). During the 30 min postinjection, the NIR‐II signal intensity measured by the same vessel in the hind limb (Figure 4d–k) illustrated a moderate blood half‐time (17.96 min as shown in Figure 4l,m), allowing for applications in delineating vascular hemodynamics under pathological status.

**Figure 5:**
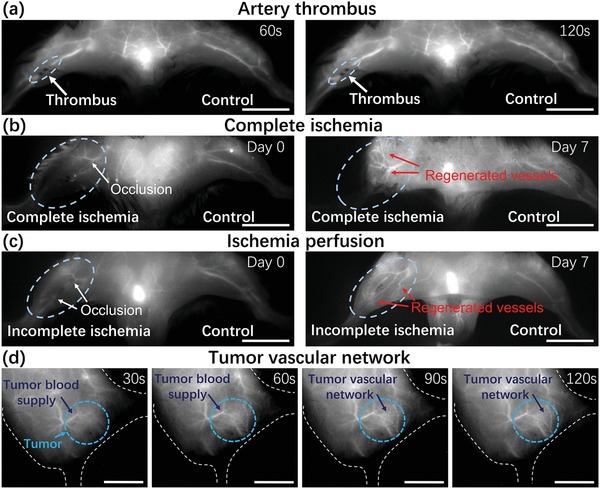
NIR‐II imaging of disorders in circulatory system. a) In vivo NIR‐II imaging of complete femoral artery thrombus (white arrows) of right hind limb at 60 and 120 s after intravenous administration (left hind limb is control). b) NIR‐II imaging of complete ischemia validated the absence of blood signal in whole hind limb (white arrows indicated the occlusion) on day 0 and regenerated vessels (red arrows) in the adjacent of the occlusion on day 7. c) NIR‐II imaging of incomplete ischemia indicated the occlusion site (white arrows) on day 0 and reconstruction of regenerated collateral vessels (red arrows) depicted the perfusion of hind limb on day 7. d) NIR‐II imaging of tumor vascular network at different time points after intravenous administration of RENPs@Lips. Scale bar: 10 mm.

Vascular stenosis or occlusion, which is usually caused by atherosclerosis or thrombosis, manifests the reduced blood supply of tissues and leads to ischemia or infarction with high morbidity.^47^, ^48^, ^49^, ^50^ In particular, acute thrombus detached from vessel leads to life‐threatening complications including myocardial infarction (MI), stroke, and pulmonary embolism (PE).^49^, ^50^, ^51^ For visualizing dysfunction of blood circulatory system preclinically, a complete femoral arterial thrombus murine model was established and used for imaging. Shortly after RENPs@Lips were injected intravenously, a significant NIR‐II signal discontinuity of blood flow was obtained as shown in **Figure** 5a, which precisely matched with the induction area of surgery. Encouraged by the fast feedback of hemodynamic with high resolution, we further established complete and incomplete ischemia of hind limb models to explore the feasibility of RENPs@Lips in identifying and monitoring the ischemia perfusion dynamically. Apparently, the ischemia range of right hind limb was easily detected in the whole proximal region of complete occlusion immediately after intravenous administration on day 0. We further dynamically traced the same model of complete ischemia on day 7 and the NIR‐II signal of proximal region to the ischemia occlusion was still absent. Besides, regenerated vessels in the adjacent of the occlusion on day 7 was clearly visualized (Figure 5b). Not surprisingly, the model mice had edema and necrosis in the proximal of right hind limb with abnormal pace on day 7. As for incomplete ischemia, large numbers of regenerated vessels with complicated patterns could be monitored dramatically, reflecting the recruitment of collateral vessels to acute remodeling and blood perfusion of whole hind limb on day 7 (Figure 5c), also consistent with the nearly normal appearance of hind limb and pace of mice. By contrast, the vasculature structure of the normal side of hind limb remained unchanged. All these results suggest that RENPs@Lips, as NIR‐II nanoprobes, are capable of timely identifying and monitoring the blood system–related disorders.

### 2.5 NIR‐II Imaging of Tumor Vessels and Surgical Navigation

**Figure 6:**
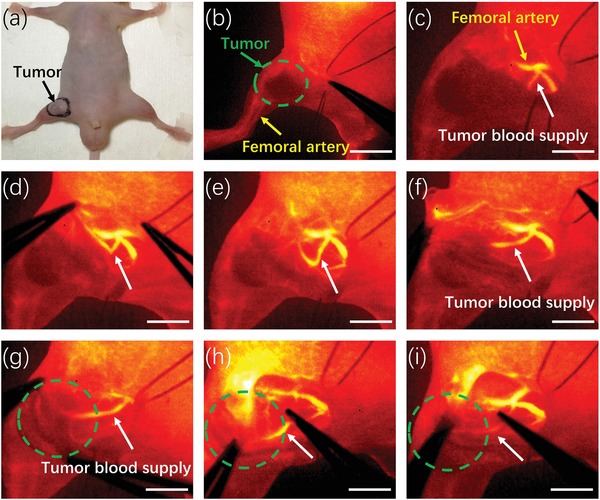
NIR‐II image‐guided intraoperative surgery. a) Digital photograph of nude mice with femur orthotopic osteosarcoma. b–g) The major blood supply of osteosarcoma was clearly discriminated and exposed from femoral artery under NIR‐II guidance. h,i) A vessel clamp was implemented to induce the temporary block of tumor major blood vessel and the signal of blood flow toward the tumor disappeared, implicating the successful accomplishment of embolization surgery of osteosarcoma. Scale bar: 4 mm.

It is well known that malignant tumor progression and metastasis are highly depended on adequate blood supply.^52^ The past decades have witnessed the numerous ascertains in antineoplastic drugs combating tumor angiogenesis.^53^, ^54^, ^55^ Therefore, the pioneering work to delineate tumor vessels can aid researchers to assess the therapeutic effects preclinically and guide surgeons to conduct embolization therapy of tumor intraoperatively. To visualize the tumor vascular network, nude mice with orthotopic xenograft 143B osteosarcoma were subsequently employed. 30 s after injection of RENPs@Lips via tail vein, the major blood supply of tumor was visible, and the complicated vascular network was gradually distinguishable during the next 90 s as shown in Figure 5d, exhibiting a typical characteristic of abundant irregularly branches. Inspired by the delineation of tumor's major blood supply, NIR‐II image‐guided surgery of femur orthotopic osteosarcoma was performed (**Figure** 6a) in order to explore intraoperative navigation for tumor vascular embolization. The major blood supply of tumor was clearly discriminated from femoral artery (Figure 6b–g). Then, a vessel clamp was implemented to block the blood flow mimicking the clinical vascular embolization procedure (Figure 6h). Dramatically, the signal of blood flow toward the tumor disappeared (Figure 6i), implicating the successful accomplishment of embolization surgery for tumor blood supply. Hence, these findings further open the possibility of RENPs@Lips for tumor vessels delineation preoperatively and accurate detection during surgery, making RENPs@Lips promising nanoprobes for the potential applications in surgical navigation of NIR‐II imaging.

### 2.6 NIR‐II Imaging of Sentinel Lymph Node Mapping and Biopsy

**Figure 7:**
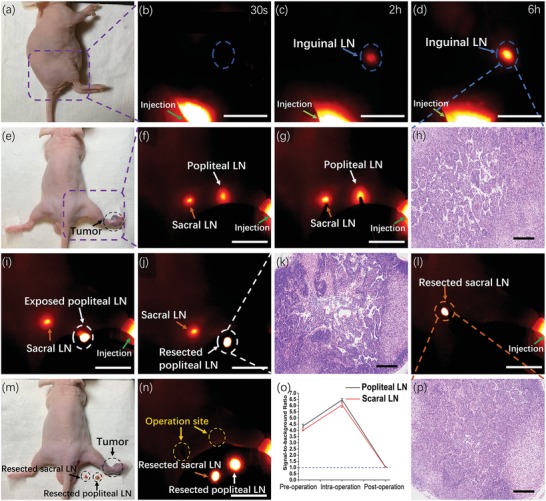
NIR‐II imaging of sentinel lymph node mapping and biopsy. a) Digital photograph of normal nude mice and b–d) inguinal lymph node (LN) was gradually visualized. e) Digital photograph of nude mice with xenograft melanoma. f,g) The popliteal LN and sacral LN were visible at 6 h postinjection. h) Histological analysis of resected inguinal LN. i,j) Resection of popliteal LN and k) histological analysis positive (+). l) Resection of sacral LN and p) histological analysis negative (−). m,n) Digital photograph and NIR‐II image of resected LNs and operation site, demonstrating the efficacy and accuracy of SLNB. o) SBR of popliteal LN and sacral LN during surgery. Scale bar: 10 mm. For histological analysis, scale bar: 200 µm.

The pathophysiological alterations of the lymphatic system are considered important diagnostic indexes in various pathological processes, especially tumor metastasis.^56^, ^57^ Accurate lymph node (LN) identification and dissection significantly decrease relapse of tumor metastasis and alleviate side effects.^58^, ^59^ Over the past several years, favorable achievement in optical imaging has been made in sentinel lymph nodes (SLNs) mapping and biopsy.^60^, ^61^ To illustrate the efficacy to localize SLN, RENPs@Lips were injected intradermally near the base of the tail of normal nude mice (*n* = 3, **Figure** 7a). During the next 6 h, the inguinal lymph node was gradually visualized with high degree of clarity while scarce fluorescence existed in adjacent areas (Figure 7b–d). Due to the outstanding SBR before surgery (SBR = 6.10), the resection of inguinal lymph node was successfully accomplished with bare normal tissue remained on it. More importantly, the SBR dropped to 1.05 after surgery, suggesting the complete resection of inguinal lymph node under NIR‐II imaging guidance, which was confirmed by the histological analysis (Figure 7h).

Ascribed to the beneficial results above, we further mimicked closer SLNB procedure in clinical practice under pathological condition on nude mice (*n* = 3) with xenograft B16F10 melanoma. After 6 h, RENPs@Lips were injected intradermally at the margin of the melanoma (Figure 7e), the sentinel lymph node and secondary lymph node were clearly visible (Figure 7f,g). With the help of NIR‐II guidance, the SLN was precisely dissected quickly (Figure 7i,j) and the histological analysis confirmed that melanoma metastasis existed in SLN (Figure 7k). We subsequently dissected the secondary lymph node (Figure 7l) and histological analysis demonstrated negative (−) (Figure 7p), implicating the dissection of SLNB was accomplished with a significant SBR (Figure 7m–o). On the one hand, deeper tissue penetration and superior photochemical property make NIR‐II fluorophore RENPs@Lips more attainable to clinical translation in SLNB. On the other hand, different from other nanoparticles, RENPs@Lips do not exhibit the optical delineation of lymphatic vessels during the whole observation postinjection, implicating that the dissection during SLNB could be more facilely facilitated for fear of luminous lymph liquid leakage to interrupt operative field.

## 3 Conclusion

In summary, we have successfully synthesized liposome‐coated NIR‐II lanthanide nanoparticles, RENPs@Lips, with high biocompatibility and attainable excretability (half‐lives of 23.0 h for liver and 14.9 h for spleen). It was worth noting that after intravenous administration, over 90% of the nanoparticle can be cleared out of body in vivo within 72 h via hepatobiliary route. Due to the moderate half‐time of blood circulation (17.96 min), RENPs@Lips display favorable feasibility to delineate the hemodynamics of vascular disorders (artery thrombosis, ischemia, and tumor angiogenesis) and monitor the reconstruction of collateral vessels in blood perfusion. In addition, RENPs@Lips demonstrate good performance in identifying orthotopic tumor vessels intraoperatively and NIR‐II image‐guided embolization surgery. Moreover, outstanding SBR allows for accomplishing identification of SLNB with tumor‐bearing mice. This work leads to our NIR‐II lanthanide nanoparticles, RENPs@Lips, with high potential for widespread applications and future translation in interdisciplinary amalgamation of research in diverse fields.

## 4 Experimental Section

*Materials*: The chemical materials such as the sodium hydroxide, yttrium(III) acetate hydrate (99.9%), oleic acid (technical grade, 90%), ammonium fluoride (≥99.99%), 1‐octadecene (technical grade, 90%), and neodymium(III) acetate hydrate (99.9%) were purchased from Sigma–Aldrich without any purification. Chol, and DPPC were purchased from Anvanti Polar Lipid, Inc. DSPE‐PEG2000 was purchased from Laysan Bio Inc. Unless otherwise mentioned, all other chemicals were purchased from Sigma–Aldrich. All solvents and chemicals were used as received.

*Animal Handling*: All vertebrate animal experiments were performed under the approval of Stanford University's Administrative Panel on Laboratory Animal Care. The Charles River company provided the 7 week old female C57BL/6 and nude mice for in vivo study. All the animals were housed at the Research Animal Facility of Stanford under the approved animal protocols. All mice were anaesthetized in a rodent anesthesia machine with 2 L min^−1^ O_2_ gas mixed with 3% isoflurane before imaging. Tail vein injection of the nanoprobes was carried with a catheter and synchronized with a camera that started continuous image acquisition simultaneously. During the time course of imaging, the mice were kept anaesthetized by a nose cone delivering 2 L min^−1^ O_2_ gas mixed with 3% isoflurane. Mice were randomly selected from cages for all experiments. All groups within study contained *n* = 3 mice.

*Toxicity Assay and Biodistribution*: The cytotoxicity assay of RENPs@Lips was examined using a standard MTT method (Sigma–Aldrich, St. Louis, USA). NIH 3T3 and RAW 264.7 cells (5 × 10^3^ cells per well) were seeded in 96‐well plates overnight and incubated with different concentrations of RENPs@Lips for 48 h. Then 10 µL of the 12 × 10^−3^ m MTT stock solution was added and incubated for 4 h. After removing medium, 200 µL of dimethyl sulfoxide (DMSO) was added to dissolve the formazan crystals' precipitates. The relative cell viability (%) was measured with a Bio‐Rad microplate reader and calculated by (*A*_sample_/*A*_blank_) × 100%. For in vivo toxicity, 1 and 7 days postinjection of the nanoprobe at different doses (200 µL, 0, 1.0, 2.0 mg mL^−1^), the mice were sacrificed, and vital organs (heart, liver, spleen, lung, and kidney) were harvested. Then, the samples were embedded in optimal cutting temperature (OCT) compound, sectioned into 6 µm slices, and stained with hematoxylin and eosin (H&E).

For in vivo biodistribution assay, the normal C57BL/6 mice (*n* = 3) were anaesthetized and then imaged under NIR‐II imaging at different time points after intravenous administration of RENPs@Lips. In addition, at 16, 48, and 72 h postinjection, the mice were sacrificed, respectively, and the organs (heart, liver, spleen, lung, kidney, brain, intestines, skin, and bone) were also imaged ex vivo under NIR‐II imaging system.

*NIR‐II Imaging of Vascular System and Surgical Navigation*: Animal models of unilateral thrombus formation and ischemia were based on a method reported previously.^62^, ^63^ Subsequently, after RENPs@Lips (200 µL, 1.0 mg mL^−1^) injection through tail vein, NIR‐II images were recorded immediately (1000 nm LP, 1000 ms). For assessment of blood perfusion and vascular regeneration in response to acute ischemia, NIR‐II images were recorded immediately on the same mouse after injection on day 0 and day 7. For intraoperation of tumor blood supply, the skin around the tumor was incised shortly after injection of RENPs@Lips. After the tumor blood vessel branch was exposed from femoral artery, the vessel clamp was employed to induce the temporary block of tumor major blood vessel.

*NIR‐II Image‐Navigated SLNB*: After being anaesthetized, RENPs@Lips (40 µL, 1.0 mg mL^−1^) were injected intradermally near the base of the tail (normal nude mice) and at the margin of tumor (nude mice with B16F10 melanoma xenograft). Then, images were recorded at different time points postinjection to visualize the SLNs. After visualizing the luminous lymph nodes, the SLNs were resected precisely under NIR‐II imaging guidance. Then the resected lymph nodes were embedded in O.C.T compound and analyzed with histological assay. If the histological results of SLNs were positive (+), the secondary LNs would be dissected. If the histological results of SLNs were negative (−), the surgery would end up.

*Statistical Analysis*: The fluorescence measurement was performed to quantitate NIR fluorescence signal intensity through the Image J 1.45× software (National Institutes of Health, Bethesda, MD). Data were given as mean ± SD (standard deviation). Statistical analysis was performed using a two‐tailed Student's *t*‐test. Statistical significance was assigned for *p*‐value < 0.05.

## Conflict of Interest

The authors declare no conflict of interest.

## Supporting information

Supplementary
