# Supplementary material for: Excretable Lanthanide Nanoparticle for Biomedical Imaging and Surgical Navigation in the Second Near‐Infrared Window
Source: Adv Sci (Weinh). 2019 Oct 4;6(23):1902042. doi: 10.1002/advs.201902042 (PMC6891904; doi:10.1002/advs.201902042)
Supplement: Supplementary file 1 — Supplementary [file ADVS-6-1902042-s001.pdf]

Copyright WILEY-VCH Verlag GmbH & Co. KGaA, 69469 Weinheim, Germany, 2019.

ADVANCED SCIENCE
Open Access

Supporting Information

for *Adv. Sci.,* DOI: 10.1002/advs.201902042

Excretable Lanthanide Nanoparticle for Biomedical Imaging and Surgical Navigation in the Second Near-Infrared Window

Daifeng Li, Shuqing He, Yifan Wu, Jianqiang Liu, Qiang Liu, Baisong Chang, Qing Zhang, Zhanhong Xiang, Ying Yuan, Chao Jian, Aixi Yu, and Zhen Cheng**

Excretable Lanthanide Nanoparticle for Biomedical Imaging and Surgical Navigation in the Second Near-Infrared Window

Daifeng Li[1,2†], Shuqing He[2,3†], Yifan Wu[1,2], Jianqiang Liu[4], Qiang Liu[2], Baisong Chang[2], Qing Zhang[2], Zhanhong Xiang[2], Ying Yuan[1], Chao Jian[1], Aixi Yu[1] and Zhen Cheng[2*]*

[†]These authors contributed equally to this work.

[*]Corresponding author: email yuaixi@whu.edu.cn, zcheng@stanford.edu.

Dr. D.F. Li, Dr. Y.F. Wu, Dr. Y. Yuan, Dr. C. Jian, Prof. A.X. Yu

Department of Orthopedics Trauma and Microsurgery, Zhongnan Hospital of Wuhan University, Wuhan, Hubei, 430071, China

E-mail: yuaixi@whu.edu.cn

Dr. D.F. Li, Dr. S.Q. He, Dr. Y.F. Wu, Dr. Q. Liu, Dr. B.S. Chang, Dr. Q. Zhang, Dr. Z.H. Xiang, Prof. Z. Cheng

Molecular Imaging Program at Stanford (MIPS), Bio-X Program, and Department of Radiology, Canary Center at Stanford for Cancer Early Detection, Stanford University, Stanford, California, 94305-5344, USA

Email: zcheng@stanford.edu

Dr. S.Q. He

Academy for Advanced Interdisciplinary Studies and Department of Biomedical Engineering, Southern University of Science and Technology (SUSTech), Shenzhen, 518055, China

Dr. J.Q. Liu

Department of Orthopedics, The Fourth Hospital of Jinan, Jinan, Shandong, 250031, China

Methods

Fabrication of rare earth doped nanoparticles: $NaYF_4$: Nd 7%@$NaYF_4$

(1) First, synthesis of $NaYF_4$: Nd 7%

The $NaYF_4$: Nd 7% core nanoparticles were synthesized according to a literature method.[1]

(2) Second, synthesis of $NaYF_4$: Nd 7%@$NaYF_4$

The $NaYF_4$: Nd 7%@$NaYF_4$ core-shell nanoparticles were synthesized according to a literature method.[1]

Fabrication of Liposome functionalized RENPs

The liposomes were made from the mixture of DPPC/Chol/DSPE-PEG2000 at a molar ratio of 77.5:20:2.5. The 50 mg lipid was dissolved in absolute 10 ml ethanol in a round bottom flask. The ethanol was evaporated with a rotary evaporator at 55 °C. Then lipid membrane was hydrated by 5.0 ml saline at 55 °C for 15 min. Finally, 10 mg/ml liposomes were obtained after sonication. RENPs was dispersed in water at the same concentration of 10 mg/ml. The RENPs and liposome suspensions were both kept in a water bath at 55 °C for 5 min and mixed at a volume ratio of 1:2 (RENPs: liposome). After sonication at 55 °C for 5 min, liposome fused with RENPs to form RENPs@Lips which was collected by centrifugation at 12000 rpm for 10 min.

Spectral characterization of RENPs@Lips

Absorbance spectra of the samples were taken on Agilent spectrophotometer UV-Vis-NIR Cary 60 spectrometer. The NIR-II fluorescence emission spectrum was captured on a home-built spectroscopy set-up by exciting RENPs and RENPs@Lips with an 808 nm laser diode with a power output of 100 mW. The excitation laser was filtered with a combination of a 900 nm short-pass filters. Samples were added to either a 1 cm path-length cuvette and the resulting emission filtered through a 1000 nm long-pass filter (Thorlabs) to reject the incident excitation laser light. The emitted fluorescence was collected by a spectrometer coupled to a cooled (-80 °C) InGaAs detector array (Princeton Instruments, NIR vana: 640).

Measuring NIR-II quantum yield

Quantum yield of RENPs@Lips were measured in a similar manner as described in previous publications. [1] Briefly, a serial dilution of five solutions of IR26 as well as RENPs@Lips with an OD<0.1 at 808 nm was measured to confirm absorbance values at 808 nm and the fluorescent emission spectrum was collected on a wavelength corrected NIR-II spectrometer in a 1 cm quartz cuvette in the manner specified above. The fluorescent emission spectrum was integrated and plotted against the OD value at 808 nm and a linear fit was applied to verify the linearity between fluorescent brightness and concentration. By comparing the slope of the linear fit between IR26 and RENPs@Lips, the quantum yield was determined based on the following supporting equation:

$$\frac{\phi \text{sample}}{\phi \text{IR26}} = \frac{I1}{A1} \text{ x } \frac{A2}{I2} \text{ x } \left(\frac{n1}{n2}\right)^2$$

Where ϕIR26=0.5% in 1, 2-dichloroethane (DCE). I was the integrated fluorescence intensity and A is the absorbance at the excitation wavelength. n1 is the refractive index of hexane (1.375), n2 is the refractive index of DCE (1.43). A=0.1-0.001 in order to ensure linear response on the intensity.

Cells and tumor models

RAW 264.7 (mouse macrophage cell), NIH 3T3 (mouse embryonic fibroblast cell), B16F10 (mouse melanoma), and 143B (human osteosarcoma) were obtained from American Type Culture Collection (Manassas, VA) and cultured in Dulbecco’s modified Eaglemedium (DMEM) containing high glucose (Gibco), supplemented with 10% fetal bovine serum (FBS) and 1 % penicillin–streptomycin in a humidified atmosphere of 5 % CO_2 at 37 °C. The 143B or B16F10 cells were harvested when reaching 80-90 % confluency (roughly 5×10^6 in 100 µl of PBS) and next were inoculated into nude mice (4 to 6 weeks old, Charles River Laboratories, USA).

Supplementary Figure

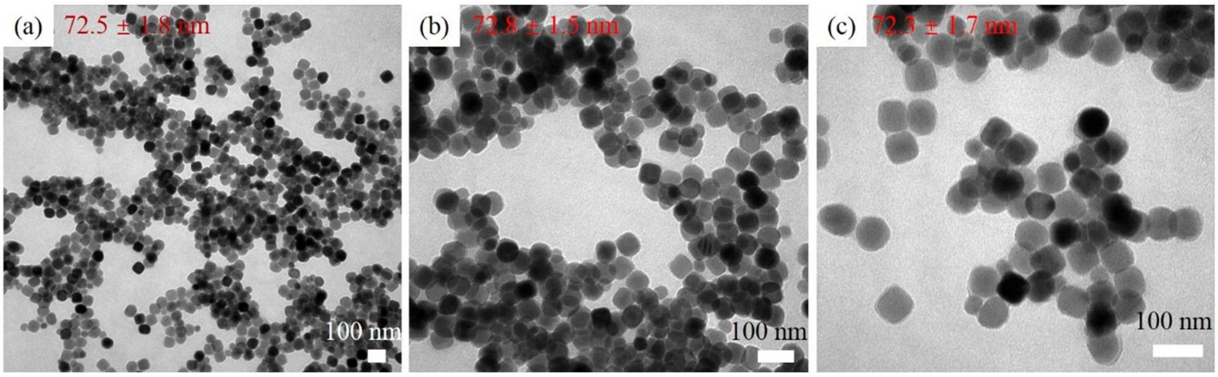

Figure S1. The low magnification TEM images of RENPs@Lips with **(a)** ×20,000 **(b)** ×50,000 and **(c)** ×80,000. Scale bar: 100 nm.

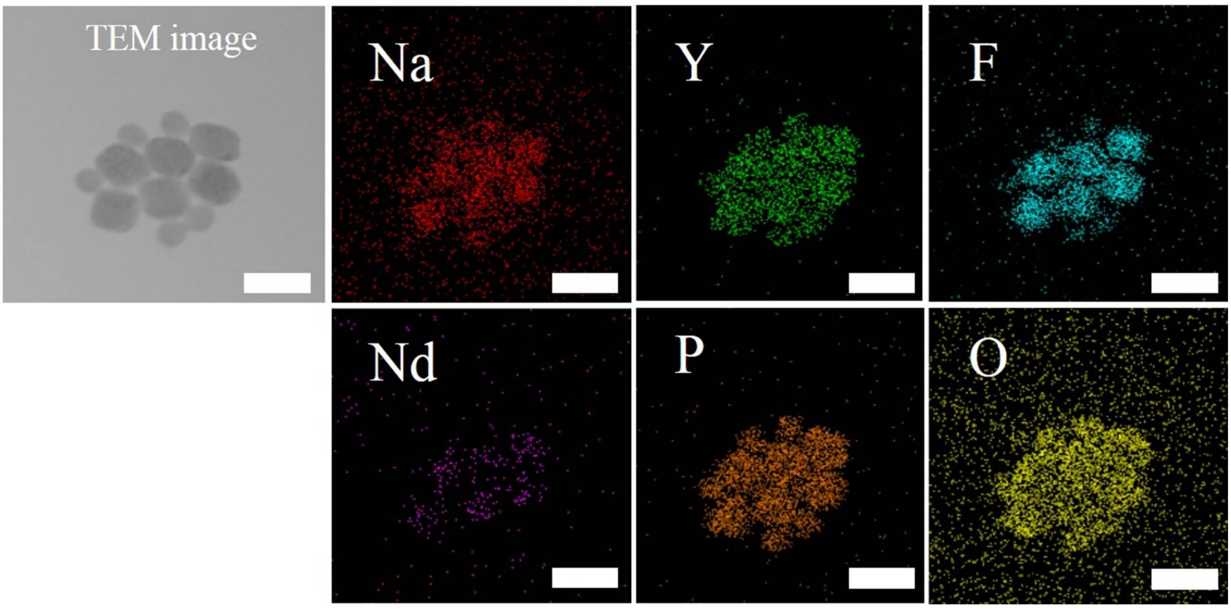

Figure S2. TEM element mapping of RENPs@Lips. Scale bar 100 nm.

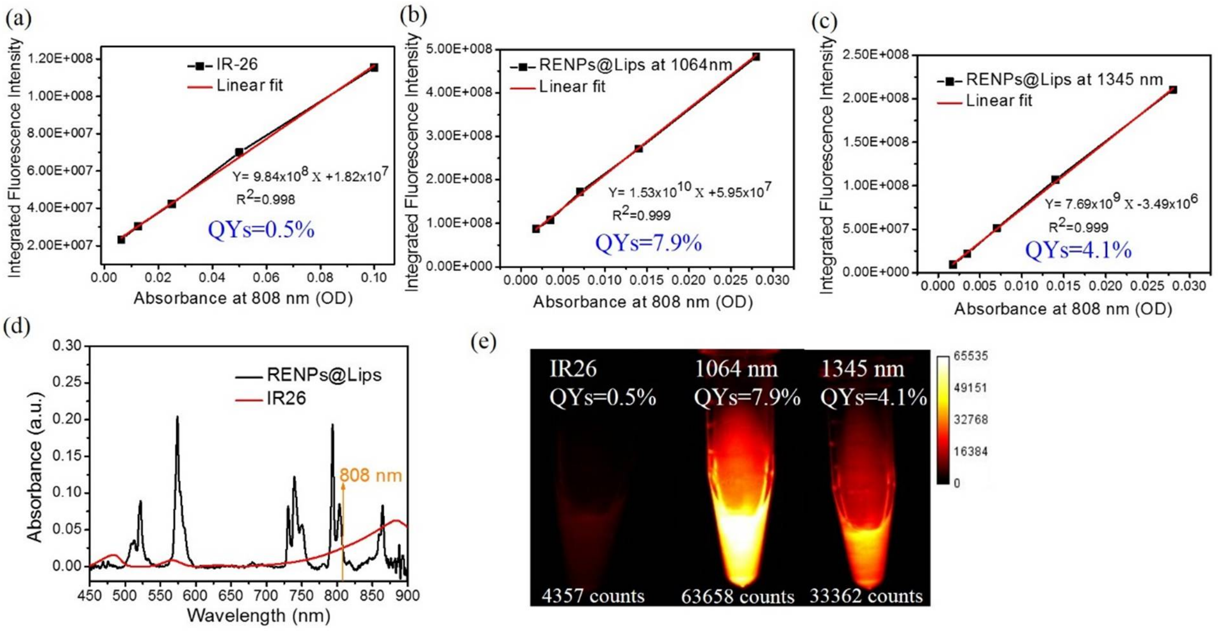

Figure S3. Quantum yields of RENPs@Lips. (a)-(c) Plot of the integrated fluorescence spectrum of IR26 and RENPs@Lips with five different concentrations (absorbance measured in OD). **(d)** the IR26 and RENPs@Lips with the same absorbance value at 808 nm. **(e)** The NIR-II imaging of IR26 and RENPs@Lips in tube with the excitation at 808 nm 0.5W/cm^2.

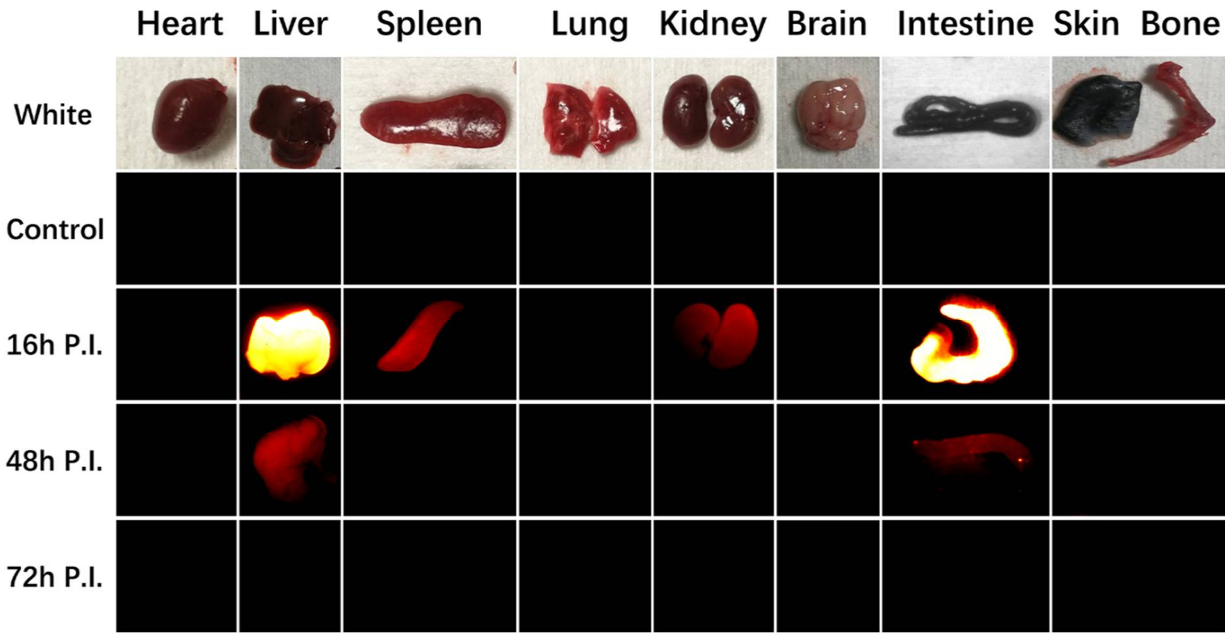

Figure S4. ***Ex vivo*** **NIR-II imaging of mice organs.** Vital organs including heart, liver, spleen, lung, kidneys, brain, intestine, skin and bone were harvested to analysis the NIR-II fluorescence intensity and biodistribution *ex vivo* at 16 h, 48 h, 72 h after intravenous administration of RENPs@Lips.

Table S1. Inductively Coupled Plasma Mass Spectrometry (ICP-MS) analysis. The ICP-MS measurement of Y^{3+} content in the corresponding organs, urine and feces demonstrated a high-level accumulation of RENPs@Lips in liver and spleen at 16 h after intravenous administration, but a sharp decline very close to the content level of control mice at 72 h after intravenous administration. In addition, the accumulation in feces 16 h after intravenous administration exhibited the main excretion of RENPs@Lips through hepatobiliary route. Notably, 96 h after intravenous administration, all the organs and feces reached to the normal level compared to the control mice, implicating the probe was excreted completely at the same concentration with the normal organs. The ICP-MS results showed high consistent with our imaging data both *in vivo* and *ex vivo*.

Tissue type n = 3	Control ng/g	16 h P.I. ng/g	72 h P.I. ng/g	96 h P.I. ng/g
Heart	17.3 ± 1.4	17.6 ± 1.8	15.9 ± 1.9	16.9 ± 1.3
Liver	20.1 ± 2.0	1505.4 ± 150.0	29.3 ± 2.6	25.0 ± 3.1
Spleen	21.6 ± 1.1	563.5 ± 12.6	21.1 ± 0.6	22.6 ± 1.9
Lung	33.7 ± 1.9	34.7 ± 2.7	32.8 ± 0.9	32.0 ± 1.6
Kidney	27.9 ± 1.1	138.0 ± 12.6	26.8 ± 1.9	27.4 ± 0.6
Bone	45.1 ± 2.2	54.0 ± 4.1	47.4 ± 1.4	47.8 ± 2.0
Urine	7.5 ± 1.2	7.8 ± 0.4	7.5 ± 1.2	7.1 ± 0.4
Feces	17.1 ± 1.7	1706.5 ± 49.9	23.6 ± 2.5	20.3 ± 4.0

Table S2. Comparison of excretability between RENPs@DSPE-mPEG and RENPs@Lips.

Nanoparticle	Composition or Modification	Half-life of blood	Half-life of liver	Half-life of spleen	Skeletal affinity
RENPs@DSPE-mPEG	β-NaYF4: Nd 7%@NaYF4<br>DSPE-mPEG	20.56 min	52.0 h	175.0 h	High
RENPs@Lips	β-NaYF4: Nd 7%@NaYF4<br>DSPE-mPEG<br>Liposome	17.96 min	23.0 h	17.9 h	Low

Reference

[1] S. He, S. Chen, D. Li, Y. Wu, X. Zhang, J. Liu, J. Song, L. Liu, J. Qu, Z. Cheng, *Nano Lett.* **2019**, *19*, 2985.
